# At thermoneutrality, acute thyroxine-induced thermogenesis and pyrexia are independent of UCP1

**DOI:** 10.1016/j.molmet.2019.05.005

**Published:** 2019-05-26

**Authors:** Claudia Dittner, Erik Lindsund, Barbara Cannon, Jan Nedergaard

**Affiliations:** Department of Molecular Biosciences, The Wenner-Gren Institute, Stockholm University, Stockholm, Sweden

**Keywords:** Thyroid hormone, Thermogenesis, UCP1, Hyperthermia, Pyrexia, Fever, Brown adipose tissue

## Abstract

**Objective:**

Hyperthyroidism is associated with increased metabolism (“thyroid thermogenesis”) and elevated body temperature, often referred to as hyperthermia. Uncoupling protein-1 (UCP1) is the protein responsible for nonshivering thermogenesis in brown adipose tissue. We here examine whether UCP1 is essential for thyroid thermogenesis.

**Methods:**

We investigated the significance of UCP1 for thyroid thermogenesis by using UCP1-ablated (UCP1 KO) mice. To avoid confounding factors from cold-induced thermogenesis and to approach human conditions, the experiments were conducted at thermoneutrality, and to resemble conditions of endogenous release, thyroid hormone (thyroxine, T4) was injected peripherally.

**Results:**

Both short-term and chronic thyroxine treatment led to a marked increase in metabolism that was largely UCP1-independent. Chronic thyroxine treatment led to a 1–2 °C increase in body temperature. This increase was also UCP1-independent and was maintained even at lower ambient temperatures. Thus, it was pyrexia, i.e. a defended increase in body temperature, not hyperthermia. In wildtype mice, chronic thyroxine treatment induced a large relative increase in the total amounts of UCP1 in the brown adipose tissue (practically no UCP1 in brite/beige adipose tissue), corresponding to an enhanced thermogenic response to norepinephrine injection. The increased UCP1 amount had minimal effects on thyroxine-induced thermogenesis and pyrexia.

**Conclusions:**

These results establish that thyroid thermogenesis is a UCP1-independent process. The fact that the increased metabolism coincides with elevated body temperature and thus with accelerated kinetics accentuates the unsolved issue of the molecular background for thyroid thermogenesis.

## Highlights

• Acute thyroxine (T4) treatment increases metabolic rate markedly, even in UCP1 KO mice.
• T4 treatment increases body temperature by 1–2 °C, even in UCP1 KO mice.
• The increased body temperature is not hyperthermia but pyrexia (a regulated increase).
• Prolonged T4 treatment recruits brown fat but this only marginally augments thermogenesis.
• UCP1 activity is not the molecular mechanism for thyroid thermogenesis.

## 1 Introduction

Hyperthyroidism is associated with increased metabolism [1], [2], [3] and an increased body temperature. The molecular nature of the increased metabolism has not been clarified as yet, although several investigations point to the possibility that the increased metabolism is due to activation of brown adipose tissue through a centrally mediated effect [4], [5], [6]. According to this tenet, thyroxine (T4) released from the thyroid gland would reach areas within the ventromedial hypothalamus, and this would result in stimulation of brown adipose tissue through activation of the sympathetic nervous system. This means that the stimulation of thermogenesis by thyroid hormone would occur in a similar manner to the stimulation induced by cold (for classical nonshivering thermogenesis) [7], [8] or food (for so-called diet-induced thermogenesis) [9]. It would thus occur through the release of norepinephrine from the sympathetic nervous system and stimulation of the brown-fat cells. In the brown-fat cells, thermogenesis would then occur due to activation of the brown-fat-specific uncoupling-protein-1 (UCP1) [7], [8]. Additionally, there is evidence for peripheral effects of thyroid hormone on the brown adipocytes themselves: the expression of UCP1 requires the presence of thyroid hormone [10] and the expression in brown adipose tissue of the deiodinase that converts thyroxine to T3 is highly positively correlated with brown adipose tissue recruitment [11], [12], [13], [14].

To assess the generality of the observations concerning centrally administered thyroid hormone for the more physiological condition of peripherally released thyroid hormone (mimicking the release from the thyroid gland), we have examined here thyroid thermogenesis induced by peripherally administered thyroxine in wildtype mice as compared to mice devoid of UCP1 (UCP1 KO mice). In the UCP1 KO mice, both brown and brite/beige adipocytes completely lose their ability to carry out adrenergically induced thermogenesis [15], [16]. The investigations were performed at thermoneutrality to avoid activation of brown adipose tissue by cold stress [17] and to better approach human conditions. We found that thyroid thermogenesis was practically independent of the presence of UCP1. Also the increase in body temperature was UCP1-independent; this elevated body temperature was thus not hyperthermia but was due to a thyroid hormone-induced increase in the defended body temperature: pyrexia.

## 2 Materials and methods

### 2.1 Animals

All experiments were approved by the North Stockholm Animal Ethics Committee. UCP1 KO mice originated from the institute's own breeding and were the descendants of those earlier described [18]; they had been back-crossed to wildtype C57Bl/6 for at least ten generations; the wildtype (C57Bl/6) mice were obtained from Nova, Germany. During experimentation, mice were housed at 30 °C with relative humidity of 45–52%, and a 12 h/12 h light–dark cycle. They were single caged and had free access to standard chow diet and water. Body weight and food consumption were measured daily. Food consumption was determined by weighing the food offered and the remaining food in the cages every day. Rectal temperature was measured using a rectal probe for mice and a BAT-12 microprobe thermometer (Physitemp Instruments, Inc. USA). These procedures were performed daily at 30 °C ambient temperature between 10 a.m. and 2 p.m.

### 2.2 Magnetic resonance imaging

The body composition of the mice was measured where indicated by magnetic resonance imaging (MRI) using a magnetic resonance scanner (Echo MRI™) for small animals. The mouse was placed in a translucent plastic tube while conscious and positioned carefully so that it did not move during the measurement. The tube was then inserted into the magnetic resonance imaging apparatus; the measurement itself took about 30 s.

### 2.3 Induction of hyperthyroidism

To induce a state corresponding to hyperthyroidism, l-thyroxine was injected daily for 3 days or 21 days as indicated. l-Thyroxine was obtained from Sigma–Aldrich (Germany) and dissolved in sterile physiological saline solution with NaOH. The pH of the final solution was 7–8. The vehicle control was the saline solution containing the same amount of NaOH. Solutions were prepared weekly and were aliquoted into daily portions and stored at 4 °C until used. l-Thyroxine was administered by subcutaneous injection (s.c.) into the back of the animal at a dose of 2 mg/kg body weight; the injection volume did not exceed 50 μl.

### 2.4 Indirect calorimetry for basal metabolic rate

The metabolic rate of the mice was measured for two nights and one day using an indirect calorimetry system (INCA) from Somedic, Sweden. In their home cages, mice were placed in a temperature-controlled chamber; air was pumped through, and oxygen consumption and carbon dioxide production data were collected every second minute. Inside the chamber, the mice had free access to standard chow diet and water, the ambient temperature was set to 30 °C, and mice experienced the same 12 h/12 h light–dark cycle as they did in the housing rooms. The mice were usually placed into the indirect calorimeter at about 5 p.m. Data from the first night were discarded as it was considered to be adaptation time to the new environment. The data recorded in the 24 h from 8 a.m. to 8 a.m. the next day were used for analysis. The experiments were performed at the end of the respective l-thyroxine treatment period.

### 2.5 Indirect calorimetry for norepinephrine stimulation

For norepinephrine stimulation, the mice were anesthetized by an intraperitoneal injection of pentobarbital at a dose of 75 mg/kg body weight. The mice were placed in the indirect calorimeter at 33 °C immediately after the anesthesia showed full effect; this high ambient temperature was used as the mice otherwise drop in body temperature during anesthesia. The metabolic rate under anesthesia was recorded for about 30 min, then 1 mg/kg body weight L-(−)-norepinephrine bitartrate salt monohydrate (Sigma–Aldrich, Germany) dissolved in physiological saline solution was injected subcutaneously behind the neck to induce thermogenesis.

### 2.6 Short-term stimulation with l-thyroxine

Short-term stimulation with l-thyroxine was performed in a total of 24 female mice, 16 wildtype (C57BL/6) and 8 UCP1 KOs; an additional 16 female wildtype and 8 female UCP1 KO mice received vehicle treatment. The treatment was started at the age of 12 weeks; wildtype mice weighed 21–27 g and UCP1 KO mice weighed 19–24 g. One week prior to treatment, animals were acclimatized to 30 °C and they were single-caged 4 days prior to treatment. The l-thyroxine treatment lasted for 3 days, and body composition was analyzed by MRI on days 1 and 3. From the evening of day 3 to the morning of day 5, metabolic rates were recorded by indirect calorimetry as described in section 2.4; afterwards the animals were stimulated with norepinephrine, as described in section 2.5. Mice were euthanized with CO_2_ while still under pentobarbital anesthesia.

### 2.7 Chronic stimulation with l-thyroxine

The chronic effect of l-thyroxine treatment on mice was evaluated in 16 female and 16 male wildtype (C57BL/6) mice and 16 female and 16 male UCP1 KO mice; half of each group received subcutaneous l-thyroxine injections, whereas the other half received subcutaneous vehicle injections. The treatment started at the age of 12 weeks and was continued for 21 days; mice weighed 20–23 g (wildtype female), 24–26 g (wildtype male), 17–23 g (UCP1 KO female), and 21–30 g (UCP1 KO male) at the start of the treatment. The mice were acclimatized to 30 °C for 9–11 days prior to treatment and single-caged 2–4 days prior to treatment. The body composition of the mice was analyzed by MRI on day 0 (one day before treatment), day 8, day 15 and day 21. From the evening of day 21 to the morning of day 23, metabolic rates were recorded by indirect calorimetry as described in section 2.4; afterwards the mice were anesthetized and then stimulated with norepinephrine as described in section 2.5. Mice were euthanized with CO_2_ while still under pentobarbital anesthesia. Interscapular brown adipose tissue (IBAT), inguinal white adipose tissue (ingWAT), and gonadal white adipose tissue (gWAT) were quantitatively dissected, weighed and snap-frozen and stored at −80 °C for further analysis.

### 2.8 Influence of ambient temperature on rectal temperature

To investigate the influence of different ambient temperatures on the rectal temperature of the mice, the mice were exposed to different ambient temperatures for 1 h starting at 1 p.m. and their rectal temperature was then measured at 2 p.m. Ambient temperatures for this experiment were thermoneutrality (30 °C), room temperature (23 °C), and 4 °C.

### 2.9 Thermoregulatory metabolic responses to an ambient temperature ramp

The mice were placed inside the indirect calorimeters in their home cages at 8 a.m. They were allowed to adapt to their new environment for 3.5 h at 30 °C; then the temperature was decreased 5 °C every 1.5 h until an ambient temperature of 5 °C was reached.

During the last 30 min at each temperature, the average of 10 measurement values obtained from resting animals was taken to represent the resting metabolic rate at that particular ambient temperature. As we have experienced that the mice tolerate higher temperatures poorly, we did not increase the ambient temperature above 30 °C.

### 2.10 Western blot analysis

For protein analysis, tissues samples were homogenized in RIPA buffer with protease inhibitor (Complete-Mini, Roche Diagnostics). IBAT was mechanically homogenized in 10 ml/g RIPA buffer and ingWAT in 3 ml/g RIPA buffer. After homogenization, the samples were centrifuged at 14 000 ×*g* for 15 min and the supernatant collected. Protein concentration was measured with Pierce™ BCA Protein Assay kit (Thermo Fisher, 23225), essentially following the manufacturer's instructions. Samples were prepared with 1:1 ratio of sample buffer (66 mM Tris–HCl pH 6.8, 73 mM SDS, 360 mM glycerol, 50 mM DTT and a very small amount of BpB) and placed at 95 °C for 5 min. To determine relative UCP1 expression, 0.4 μg thyroxine-treated IBAT, 2 μg vehicle-treated IBAT and 10 μg ingWAT were loaded onto a 12% SDS-polyacrylamide gel. To quantify between membranes, 2 μg of an internal standard, consisting of pooled IBAT from several mice, was loaded on each membrane. Following electrophoresis, the protein was transferred, via electroblotting, to a polyvinylidene difluoride membrane. The membrane was blocked in 5% low-fat milk and incubated with UCP1 polyclonal antibodies (rabbits inoculated with the mouse UCP1 C-terminal decapeptide) and anti-rabbit IgG HRP-linked antibody (Cell Signaling, 7074); the primary antibody was diluted 1:12 000 in 5% BSA and the secondary antibody was diluted 1:12 000 in 2.5% low-fat milk. Chemiluminescence was detected in a CCD camera (Fujifilm) with detection reagent (Clarity Western ECL Substrate, BioRad). Ponceau S (0.1% with 5% acetic acid) was used for loading control (not shown). The samples were analyzed blinded; samples closest to the mean for each group were selected for a display membrane. For quantification, the internal standard was set to 1 AU. Quantification was performed with Image Gauge 3 software. All values are expressed as mean ± standard error.

### 2.11 Statistical analysis

Statistical analysis was performed with Prism, using Student's two-tailed non-paired t-test. P values below 0.05 were considered statistically significant. To estimate the statistical uncertainties of values calculated as differences between means, the SD was calculated in quadrature (i.e. as the square root of the sum of the ingoing squared SDs). The SD values thus obtained were also used for calculations of statistical significances of these differences between means.

## 3 Results

### 3.1 Thyroxine markedly increases metabolic rate independently of UCP1

To evaluate the significance of brown adipose tissue and particularly that of UCP1 for the metabolic effects of thyroid hormone, we investigated mice acclimated to thermoneutrality. In this way, confounding effects of the cold exposure associated with standard mouse housing at 20 °C are avoided, and these conditions better approach human metabolic conditions, since humans effectively rarely encounter prolonged cold stress [17].

Although T3 is the genomically active form of thyroid hormone, we used treatment with thyroxine (T4) rather than with T3. In this way, the analysis included components of the peripheral deiodinase system that metabolizes T4 to T3. This is of particular interest in relation to brown adipose tissue and UCP1 involvement in the metabolic response to thyroid hormone, since brown adipose tissue expresses high levels of deiodinase 2, and because the expression and activity of this enzyme is highly correlated with the recruitment and activity state of the tissue [11], [12], [13], [14].

To ensure that the metabolic effects of thyroxine were fully induced, we used thyroxine doses that were intended to be fully saturating for the thyroid hormone receptor and thus were higher than the doses used for re-establishing a euthyroid state in hypothyroid animals. It should be noted that these mice are not formally hyperthyroid in that they do not have an increased thyroid hormone level due to overactivity of their thyroid gland but due to the thyroxine treatment. Instead, these mice present with thyrotoxicosis, i.e. the physiological state induced by an elevated level of thyroid hormone, irrespective of whether the thyroid hormone is of endogenous or exogenous origin (although these terms are occasionally used interchangeably). We examined these mice versus normal, i.e. euthyroid, mice.

#### 3.1.1 Short-term thyroxine treatment

**Figure 1:**
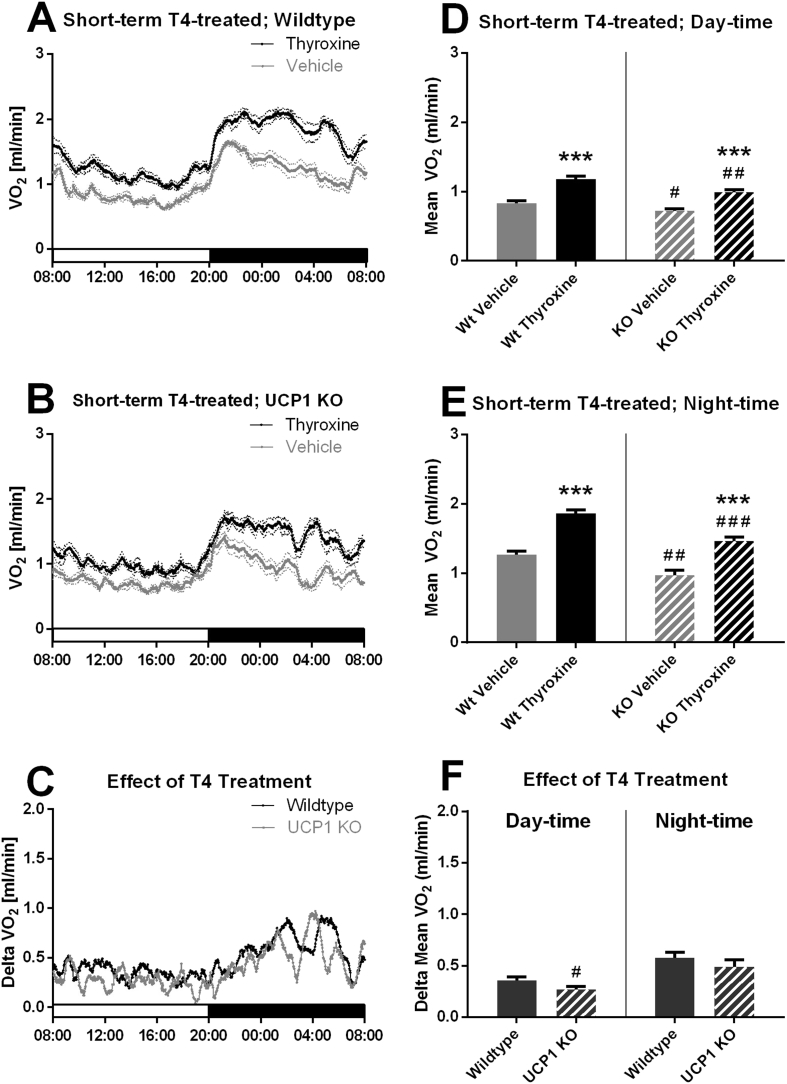
**The increased metabolism induced by short-term treatment of mice with thyroxine is UCP1 independent.** Wildtype and UCP1 KO female mice were treated for 3 days with l-thyroxine and their oxygen consumption followed in a metabolic chamber during the 4th day. A. *Metabolic rate wildtype.* Values are means ± SE; n = 14/14 (vehicle/thyroxine). B. *Metabolic rate UCP1 KO*. n = 8/8 (vehicle/thyroxine). C. *Effect of thyroxine on metabolic rate.* Values are calculated for each time point as (mean vehicle-treated minus mean thyroxine treated). D and E. *Mean metabolic rate day-time and night-time.* Values are means ± SE. F. *Effect of thyroxine on mean metabolic rate day-time and night-time.* Values calculated as mean vehicle-treated minus mean l-thyroxine treated. Statistical uncertainty was estimated as indicated in Methods. Here and in the following graphs, *, ** and *** indicate a statistically significant effect of thyroxine treatment within the genotype (P < 0.05, <0.01 and P < 0.001, respectively. Similarly, #, ## and ### indicate statistically significant differences between wildtype and UCP1 KO within the treatment.

To be able to distinguish between direct and adaptive effects, we first examined the more acute metabolic effects of thyroid hormone treatment. When examined on the fourth day, wildtype mice that were permanently housed at thermoneutral temperature and treated peripherally with thyroxine (T4) (2 mg/kg) for three days showed a marked increase in metabolic rate throughout the light/dark cycle (Figure 1A), with no effect of thyroxine on the underlying circadian rhythm. This is principally as expected [19], [20], although few earlier studies have been performed at thermoneutrality, and confounding effects of alterations in heat loss etc. in earlier studies could have influenced the metabolism observed (as earlier discussed in other contexts [21]).

Notably, mice with an ablation of the UCP1 gene showed an increase in metabolism of similar magnitude to that observed in wildtype mice (Figure 1B), in itself demonstrating that UCP1 activity is at least qualitatively dispensable for the hypermetabolism induced by our thyroxine treatment. Subtraction of the metabolic rates of the vehicle-treated mice from the thyroxine-treated mice allowed for an evaluation of the effect of thyroxine on metabolic rate in the two genotypes (Figure 1C). Clearly, the major effect of thyroxine treatment on metabolic rate occurred during the night, the active phase. There appeared to be only a marginal difference between the wildtype and the UCP1 KO mice. Average metabolic rates calculated for the whole of the light as well as the dark cycles are shown in Figure 1D,E. While it was again clear that thyroxine caused a marked increase in metabolic rate, there appeared to be only a minor – if any – effect of ablation of the UCP1 gene on the metabolic effect of thyroxine (Figure 1F).

#### 3.1.2 Chronic thyroxine treatment

**Figure 2:**
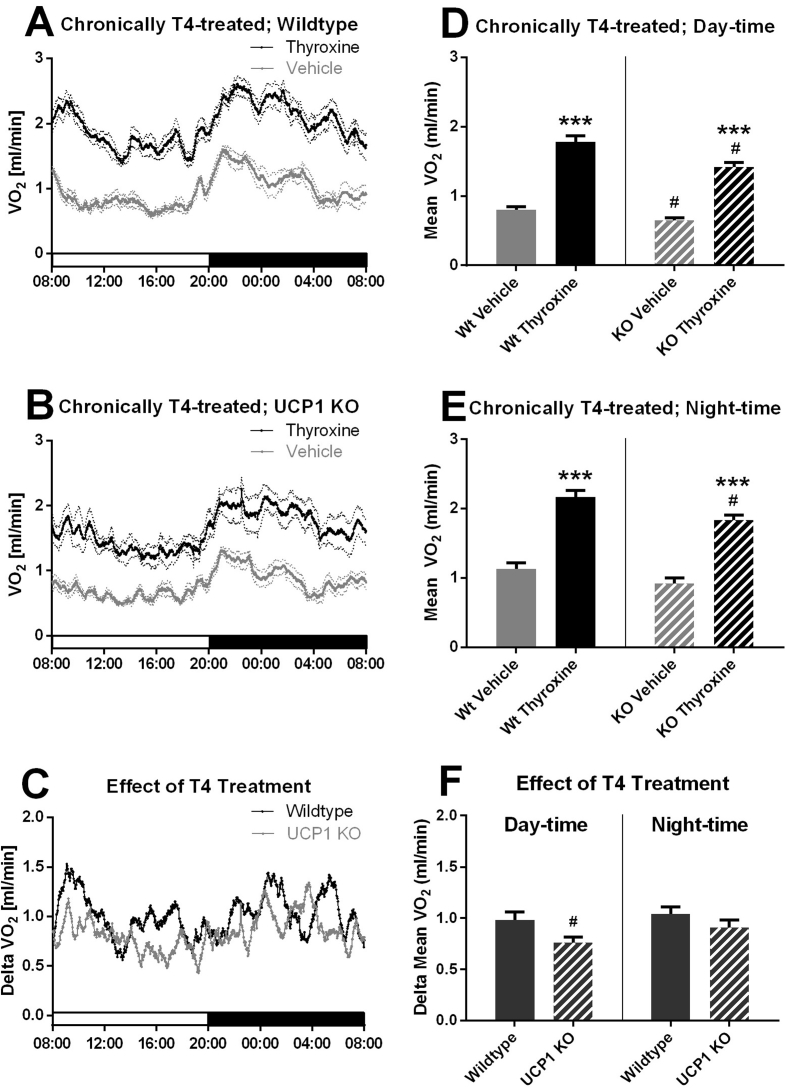
**Increased metabolism induced by long-term treatment of mice with thyroxine is mainly UCP1 independent.** Wildtype and UCP1 KO female mice were treated for 21 days with thyroxine and their oxygen consumption followed in a metabolic chamber during the 22nd day. A. *Metabolic rate wildtype.* Values are means ± SE; n = 7/8 (vehicle/thyroxine). B. *Metabolic rate UCP1 KO.* n = 8/6 (vehicle/thyroxine). C. *Effect of thyroxine on metabolic rate.* Values are calculated for each time point as (mean vehicle-treated minus mean thyroxine treated). D and E. *Metabolic rate day-time and night-time.* Values calculated as mean vehicle-treated minus mean l-thyroxine treated. F. *Effect of thyroxine on metabolic rate day-time and night-time.* Values calculated as mean vehicle-treated minus mean l-thyroxine treated. Statistical uncertainty was estimated as indicated in Methods. All figure statistics as in Figure 1.

While the above results indicated that UCP1 was dispensable for the more acute thyroxine-induced increase in metabolism, the treatment was only for three days, and full recruitment of UCP1 takes weeks [7]. As the mice were housed at 30 °C, even the wildtype mice would be expected to possess only minor amounts of UCP1 [22] and thus the contribution – if any – of UCP1 would be small. There are reports that even in euthyroid animals, thyroxine can increase UCP1 expression [20], [23], [24]. This being the case, a more prolonged treatment with thyroxine may result in UCP1 amounts of sufficient magnitude to significantly influence the metabolic response to thyroxine. The mice were therefore treated peripherally with the same daily dose of thyroxine as before but now for three weeks. Wildtype mice showed a dramatic increase in metabolic rate as a consequence of the prolonged treatment, notably greater than in the short-term treatment and resulting in as much as a doubling of their metabolic rate, again without any effect on the underlying circadian rhythm (Figure 2A). The UCP1-ablated mice also showed a very marked increase in metabolic rate, which was also notably greater than that in the short-term treated mice (Figure 2B). The calculated effect of thyroxine on metabolic rate can be seen in Figure 2C. This effect did not now show marked circadian variation, but the thyroxine effect appeared to be somewhat smaller in the UCP1 KO than that in the wildtype mice. Average metabolic rates during the light and dark phases were calculated as above (Figure 1D,E) and are shown in Figure 2D,E. Calculation of the thyroxine effect on the averaged metabolic rates shows that chronic thyroxine treatment clearly led to greater increases in metabolic rates than the acute treatment (Figure 2F versus Figure 1F) in both wildtype and UCP1-ablated mice and both during day-time and night-time (all P ≤ 0.001). Ablation of the UCP1 gene did result in a somewhat reduced response to thyroxine treatment during the light phase, but the difference did not reach statistical significance during the night phase (Figure 2F).

### 3.2 Effect of chronic thyroxine treatment on body weight gain and food intake

**Figure 3:**
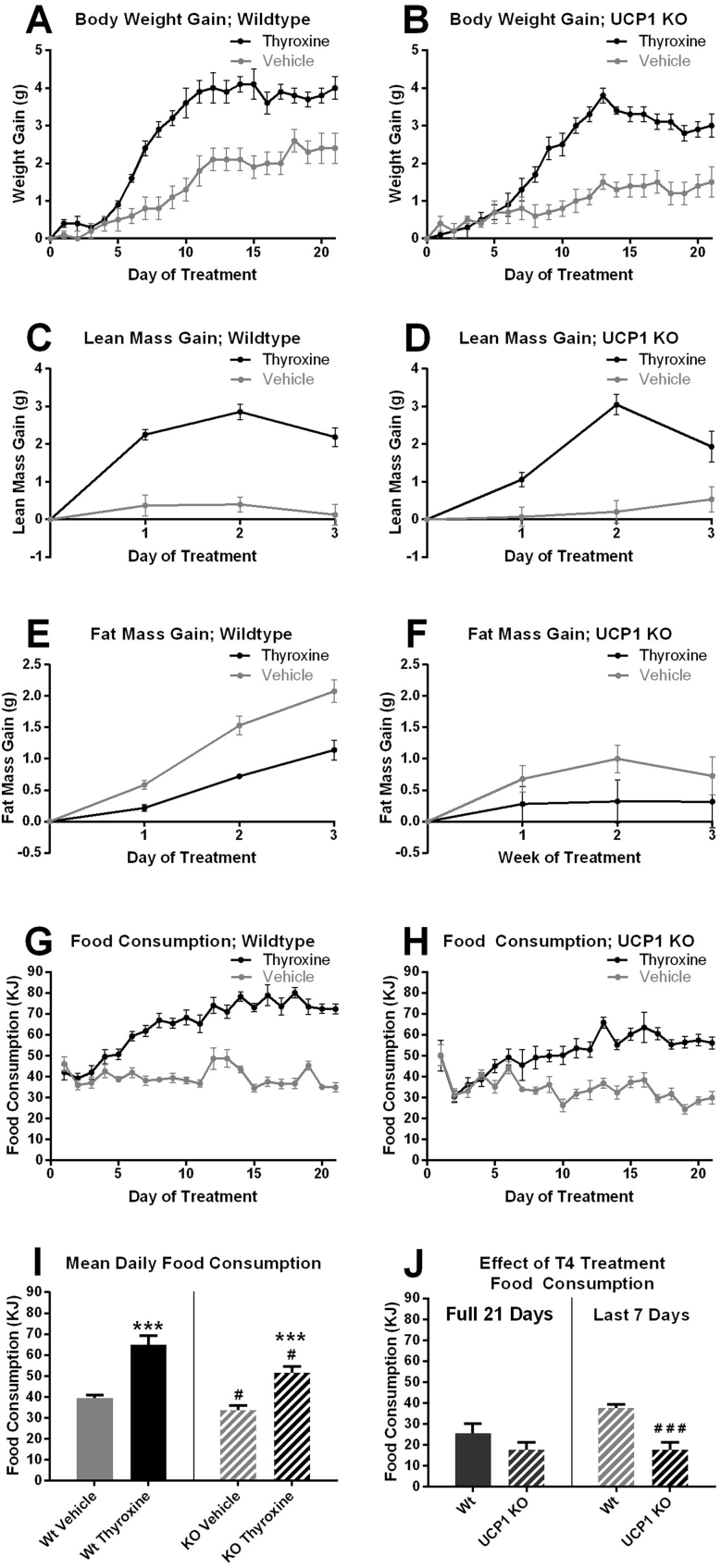
**Body weight and body composition changes and food consumption during chronic thyroxine treatment.** Data for the chronically thyroxine-treated mice metabolically characterized in Figure 2. A and B. *Body weight gain.* Means ± SE; wildtype: n = 8/8 (vehicle/thyroxine); UCP1 KO n = 8/8 (vehicle/thyroxine) for all parts of this figure. C and D. *Body fat gain, and* E and F, *Lean body mass gain.* G and H. *Daily food consumption.* I. *Average food consumption* during day 1–22. J. *Effect of thyroxine treatment on daily food consumption* day 1–22 (left) and last 7 days (right) calculated as mean value thyroxine-treated minus mean value vehicle-treated; statistical uncertainty was estimated as indicated in Methods.

Since the thyroxine-treated mice demonstrated markedly elevated levels of metabolism, this would be expected to result in decreases in body weight and/or increases in food intake. These parameters were measured during the three-week treatment period in both genotypes. It can be seen in Figure 3A,B that – perhaps in contrast to expectations in a hyperthyroid-simulating state – both groups of thyroxine-treated mice slightly (1–2 g) increased body weight to about the same extent and by at least twice the amount that the vehicle-treated mice increased. This was mainly due to an apparent increase in lean mass gain (Figure 3C–F). However, there were very marked increases in food intake in the mice of both genotypes, such that at the end of the experiment, the thyroxine-treated mice from both groups consumed approximately double as much food as the vehicle-treated mice (Figure 3G,H), which would be necessary to compensate for the vastly increased metabolic rate (Figure 2). The UCP1 KO mice had a somewhat lower thyroxine-induced intake of food, particularly during the last week (Figure 3I,J), consistent with their somewhat lower metabolic rate (Figure 2).

In both genotypes, the extra food intake would in itself explain some fraction of the increase in metabolism, due to obligatory diet-induced thermogenesis. Based on established values for the metabolic cost of processing lipid, carbohydrate and protein (2.5, 7.5 and 25 energy%, respectively) [25], the composition of the diet (10, 70 and 20 energy%, respectively) and a calorific equivalent of oxygen consumed of 20 J per ml O_2_, (cf. [26], [27]), the obligatory thermogenesis would amount to about 10% of the increased metabolism.

The effect of thyroxine on daily food intake was equivalent to an extra 2–3 g chow per day (≈28 to ≈ 38 kJ). Since much of this extra food probably remained in the digestive tract at the time of body composition measurement, this extra food may explain the apparent increase in “lean” mass, rather than thyroxine itself having led to such an increase. This would also be an explanation for the apparent body weight increase observed in Figure 3A,B.

### 3.3 Chronic thyroxine treatment elevates body temperature

**Figure 4:**
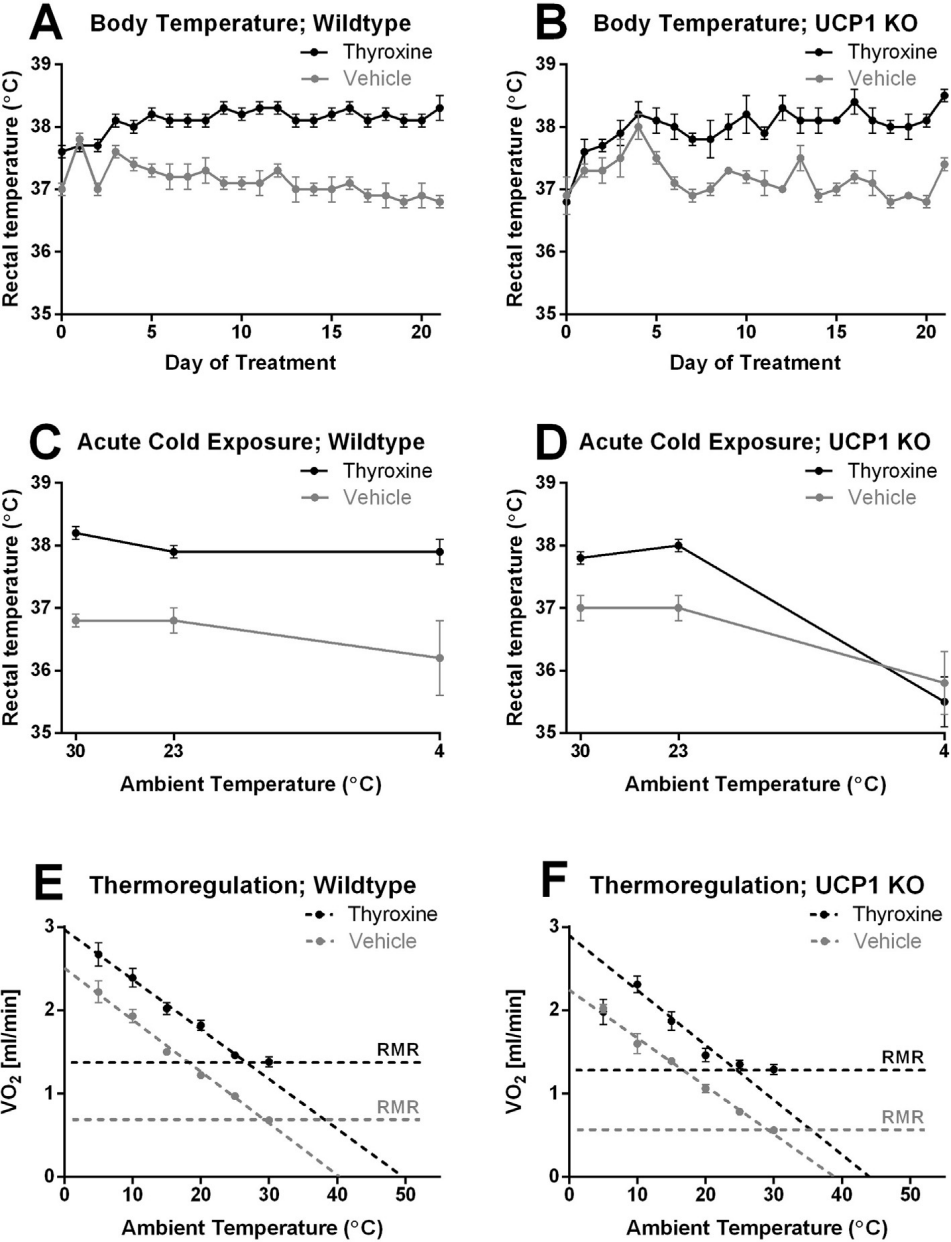
**Increased body temperature due to thyroxine treatment is UCP1 independent.** The mice metabolically characterized in Figure 2, Figure 3 were thermoregulatorily investigated. A and B. *Body temperature.* Values are means ± SE of rectal temperature measured daily. Means ± SE; wildtype: n = 8/8 (vehicle/thyroxine); UCP1 KO n = 4/4 (vehicle/thyroxine). C and D. *Acute influence of ambient temperature on body temperature.* Some of the mice in the above experiment were acutely exposed to the indicated ambient temperatures for 1 h and their rectal temperature then measured; mean values ± SE. Wildtype: n = 8/7 (vehicle/thyroxine); UCP1 KO n = 4/4 (vehicle/thyroxine). E and F. *Thermoregulatory metabolic response to ambient temperature changes.* The mice in the above experiment were acutely successively exposed to the indicated ambient temperatures and their metabolism measured; values are mean values ± SE. Wildtype: n = 8/8 (vehicle/thyroxine); UCP1 KO n = 4/4 (vehicle/thyroxine). The lines drawn are best linear fit to the values 5 °C–30 °C for wildtype vehicle, 5 to 25 for wildtype thyroxine-treated, 5 to 30 for UCP1 KO vehicle and 10 to 25 for UCP1 KO thyroxine-treated. Note that the UCP1 KO thyroxine-treated value for 5 °C coincides with that for vehicle. The horizontal lines indicated as RMR (resting metabolic rate) are visualizations based on the metabolic rate at 30 °C.

Hyperthyroidism in wildtype animals is generally associated with elevations in body temperature (e.g. [28]). In accordance with this, chronic thyroxine treatment of both wildtype mice and UCP1 KO mice resulted in body temperatures that were more than 1 °C higher than those of vehicle-injected mice (Figure 4A,B).

Interpretation of the elevated body temperature can be complex. The mice were housed at thermoneutrality and thus could not easily dispose of excess metabolic heat to the surroundings. In the absence of a simple means to dispose of heat, the thyroxine-induced increase in metabolism could therefore lead to hyperthermia, i.e. an undesired and undefended increase in body temperature. Alternatively, the higher body temperature could be the consequence of a thyroxine-induced change in the regulated body temperature (“set-point”), making the metabolic increase necessary to maintain (or be a consequence of) the new “desired” body temperature; the increased body temperature could then be considered a pyrexia (a defended elevation of body temperature, i.e. a fever not induced by an infection) (for a discussion of the terminology of body temperature regulation, see e.g. [29]).

To distinguish between these possibilities, the mice were exposed to lower ambient temperatures, such that they would be able to easily dispose of excess heat and therefore avoid hyperthermia. The wildtype results are seen in Figure 4C. It is evident that the mice defended their higher body temperature at 23 °C, in spite of the fact that they could now readily dispose of heat to the environment through vasodilation. Even when exposed to severe cold at 4 °C, the higher body temperature of thyroxine-treated mice was defended. Thus, these experiments established that the increased body temperature was not hyperthermia caused by the increased thermogenesis, because even when heat could easily be disposed of, the mice defended the higher body temperature, i.e. it was pyrexia.

The thyroxine-treated UCP1 KO mice also defended their high body temperature at 23 °C, also indicating that the higher body temperature was not hyperthermia (Figure 4D). For both groups of UCP1 KO mice, the 4 °C exposure led to a marked decrease in body temperature. This was most likely due to an inability of the mice, in the absence of UCP1-mediated thermogenesis, to generate enough heat to defend the desired, elevated body temperature (c.f. [30]).

To further analyze the question as to whether the elevated body temperature is hyperthermia or pyrexia, total energy expenditure was measured in the mice over a range of ambient temperatures, allowing construction of so-called Scholander curves that provide information about insulation and defended body temperature. The results of these measurements are given in Figure 4E,F. For the vehicle-treated wildtype mice (Figure 4E), energy expenditure increased as expected linearly with decreasing ambient temperature. Theoretically, this line would extrapolate to the defended body temperature that is 37 °C according to Figure 4A; in reality, the line extrapolated to about 3 °C above this. For the thyroxine-treated wildtype mice, the energy expenditure was elevated above that in the vehicle-treated mice at all ambient temperatures. The curve was essentially shifted upwards and in parallel with the vehicle-treated curve, extrapolating to a higher defended body temperature, also higher than the measured body temperature but clearly qualitatively in the direction that would be expected if the increased metabolism was pyrexia rather than hyperthermia (where they would extrapolate to the same temperature). This demonstrates that the thyroxine-treated animals were indeed defending a higher body temperature than the wildtype mice, showing that they were pyrexic and not hyperthermic.

It may also be discerned from the graph that the thyroxine treatment allowed them to have an extended thermoneutral zone down to about 25 °C. Since the curves for the increases in energy expenditure as a consequence of the decreasing ambient temperature were practically parallel, it can also be concluded that there were no changes in insulation caused by the thyroxine treatment.

For the vehicle-treated UCP1 KO mice, a similar curve to that for the wildtype mice was obtained: energy expenditure increased with decreasing ambient temperature (Figure 4F). The thyroxine-treated UCP1 KO mice elevated their metabolism until the ambient temperature reached 10 °C but after that failed to be able to elevate it more; in fact, it decreased, corresponding to the fall in body temperature (cf. Figure 4D). Thus, basically, both wildtype and UCP1 KO mice responded to decreasing ambient temperature by increasing metabolism, and thyroxine treatment shifted the curves upwards in parallel. The increased metabolism was therefore not due to an increased heat loss.

### 3.4 Norepinephrine-stimulated increase in metabolic rate

That the metabolic response to the chronic thyroxine treatment was greater than that to the three-day treatment could imply that there had been a recruitment of thermogenic capacity as a result of the prolonged treatment. Thermogenesis can be induced through non-adrenergically or adrenergically induced mechanisms. Adrenergic thermogenic capacity can be evaluated by measuring the metabolic response to an injection of norepinephrine. Brown adipose tissue thermogenesis is primarily mediated by norepinephrine released from sympathetic nerves innervating the tissue. A bolus injection of norepinephrine can therefore mimic local nervous stimulation of the tissue but will, of course, also activate all cells in the body that are responsive to norepinephrine. The UCP1-mediated response can be estimated as the difference between the maximal thermogenic response in wildtype mice minus the maximal response in UCP1 KO mice. The mice are anesthetized in these procedures so as to prevent activity changes from blurring the responses.

#### 3.4.1 Norepinephrine response in short-term thyroxine-treated mice

**Figure 5:**
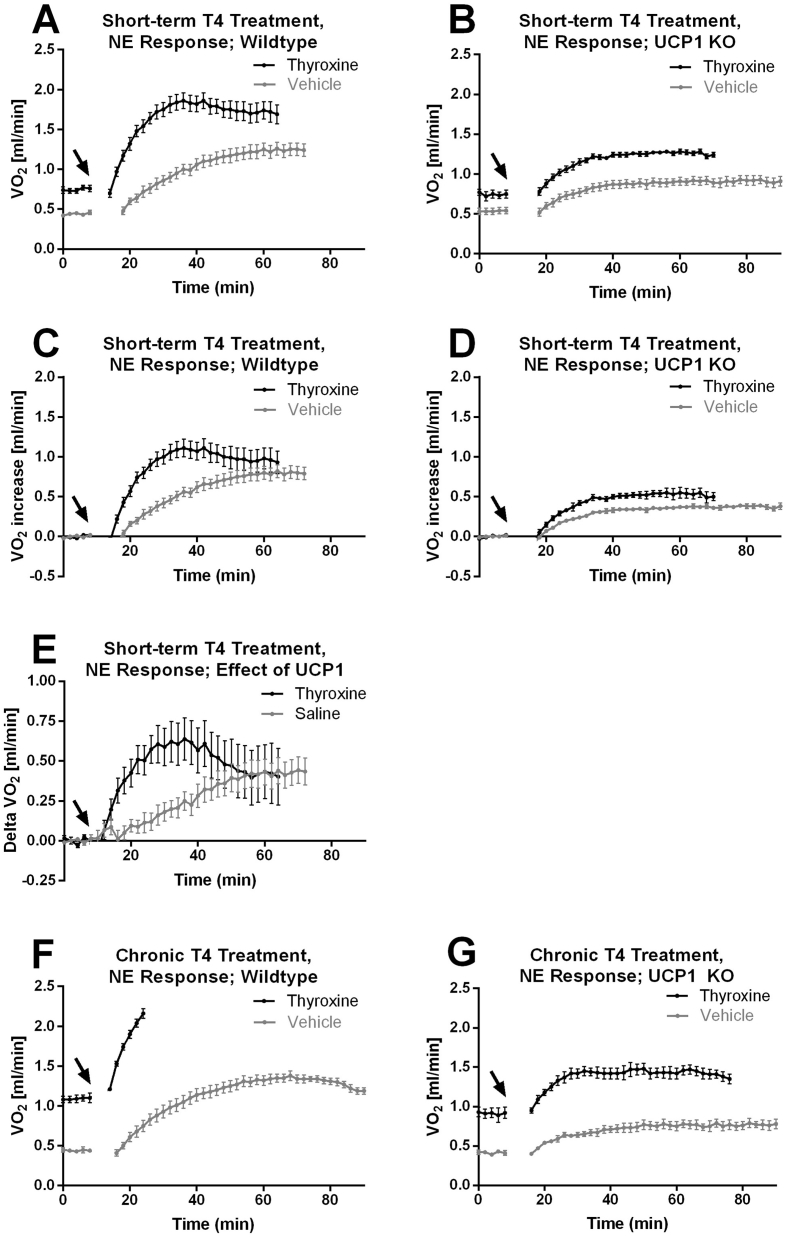
**Norepinephrine stimulation of metabolism following short-term and chronic thyroxine treatment.** A–D. The mice treated for 3 days with thyroxine the metabolic characteristics of which were shown in Figure 1 were examined for adrenergic thermogenesis capacity on the 5th day. A and B. Total oxygen consumption under anesthesia followed by norepinephrine (NE) stimulation (black arrow). Mean values ± SE. Wildtype n = 8/9 (vehicle/thyroxine); UCP1 KO n = 8/6 (vehicle/thyroxine). C and D. Norepinephrine-induced oxygen consumption. For each mouse, the basal values in A and B were subtracted from the data yielding the adrenergically induced oxygen consumption. E. UCP1-dependent response to norepinephrine injection, calculated as the mean values in D subtracted from the corresponding mean values in C; uncertainties calculated as described in Methods. F and G. The mice treated for 22 days with thyroxine the metabolic characteristics of which were shown in Figure 2, Figure 3, Figure 4 were examined for adrenergic thermogenesis capacity on the 24th day. Mean values ± SE. Wildtype n = 7/8 (vehicle/thyroxine); UCP1 KO n = 7/5 (vehicle/thyroxine).

The increase in oxygen consumption resulting from norepinephrine (NE) stimulation of the mice that had been treated for three days with thyroxine is presented in Figure 5A,B. When the thyroxine-elevated resting metabolic rate (that even during anesthesia was higher in the thyroxine-treated mice) is subtracted from the NE response (Figure 5C,D), it is seen that the predominant effect of thyroxine in the wildtype mice was an apparent change in kinetics (Figure 5C). In the UCP1 KO mice, NE injection induced only a modest increase in thermogenesis, as expected since the UCP1-dependent component was absent; the magnitude was somewhat higher in the thyroxine-treated mice (Figure 5D). Subtraction of the response in the UCP1 KO mice from the response in the wildtype mice results in the UCP1-dependent response (Figure 5E). It is clear that the kinetics are much more rapid in the thyroxine-treated mice, but the magnitude of the response is only marginally higher in the thyroxine-treated mice.

#### 3.4.2 Norepinephrine response in chronically thyroxine-treated mice

Norepinephrine-injection experiments similar to those in the short-term thyroxine-treated mice also were performed after chronic thyroxine treatment (Figure 5F,G). There was a very marked effect of thyroxine-treatment on the kinetics of the response to norepinephrine in the wildtype mice, indicating a very rapid and high heat production rate (Figure 5F). The mice did not tolerate the treatment and the experiment had to be terminated. The response recorded is thus an underestimate of the real thermogenic potential that thus could not be determined.

Also in the UCP1 KO mice there was a higher maximal response to norepinephrine in the thyroxine-treated group than in the vehicle-treated mice (Figure 5G). Although this may be interpreted as an indication of a recruitment of a (novel) UCP1-independent adrenergic thermogenic mechanism, it may be remembered that the thyroxine-treated mice maintained a higher body temperature than the euthyroid mice; the augmented response to norepinephrine injection is likely an effect of the higher temperature as such.

### 3.5 Changes in adipose tissues following thyroxine treatment

**Figure 6:**
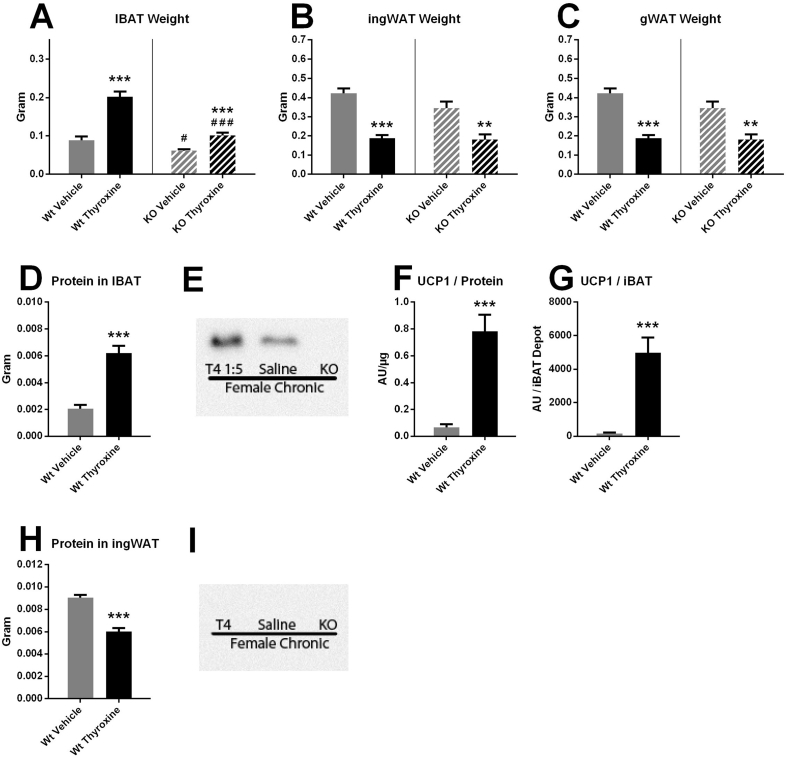
**Brown adipose tissue recruitment due to chronic thyroxine treatment.** At the end of the experiment, the adipose tissues of the mice characterized in Figure 2, Figure 3, Figure 4, Figure 5E-H were investigated. A–C. *Adipose tissue wet weights.* A: interscapular brown adipose tissue (IBAT) B: inguinal white adipose tissue (ingWAT). C: gonadal white adipose tissue (gWAT). Wildtype n = 7/8 (vehicle/thyroxine); UCP1 KO n = 8/6 (vehicle/thyroxine). Means ± SE. D. *Total protein per IBAT depot.* Wildtype: n = 7/8 (vehicle/thyroxine). E. *Western blot for UCP1 in IBAT.* F. UCP1 protein levels per mg protein in the IBAT of all wildtype mice, treated with vehicle or thyroxine. Wildtype: n = 7/8 (vehicle/thyroxine). G. Total UCP1 protein per IBAT depot. Values obtained by multiplying the total protein values (D) by the UCP1 per mg protein levels (F). H. Western blot for UCP1 in ingWAT. Positive control for UCP1 is seen in Figure 7U (same membrane).

After the chronic thyroxine treatment, the interscapular brown adipose tissue depot in the wildtype mice was very markedly increased in size (Figure 6A). In the UCP1 KO mice, similar but somewhat smaller changes were observed. In the white adipose tissue depots of both genotypes, the effect of thyroxine treatment was the opposite, that is a lower wet weight (Figure 6B). Thus, although food intake was increased considerably during thyroxine treatment, this was not sufficient to allow for the same degree of lipid storage in the white adipose depots as in the euthyroid state, indicating a very high energy consumption.

### 3.6 UCP1 levels were greatly augmented following chronic thyroxine treatment

The increase in total IBAT wet weight could either be due to lipid replenishment in the tissue (in which case the total protein amounts in the tissue would only be expected to increase marginally) or it could be due to a recruitment of the tissue, in which case protein levels should also markedly increase. In fact, total protein levels in the IBAT were nearly tripled (Figure 6D). As this indicated a recruitment due to thyroxine treatment, we examined the levels of UCP1 in the wildtype mice. We found that they were increased when analyzed per mg protein in western blots (exemplified in Figure 6E), statistically more than 10-fold (Figure 6F). After taking into account the increase in total IBAT protein, the total amount of UCP1 in IBAT was increased more than 30-fold (Figure 6G). Although the value in the untreated mice, as a consequence of thermoneutrality and chow diet, presumably represents the lowest physiologically obtainable UCP1 levels, the increase is nonetheless such that it should convey some significant thermogenic capacity to the BAT (Figure 5F). This higher capacity is probably the background to the observation that the thyroxine-treated mice did not tolerate the norepinephrine injection under thermoneutral conditions. Therefore, it is clear that chronic thyroxine treatment indeed led to increased UCP1 protein expression in brown adipose tissue and therefore provided a possibility for a greater contribution from nonshivering thermogenesis to the elevated metabolism of the thyroxine-treated mice.

Western blot analysis was also carried out on the inguinal white adipose tissue samples from all groups of mice; in these samples, no UCP1 protein was detectable (Figure 6I).

### 3.7 The effect of chronic thyroxine treatment in male mice

**Figure 7:**
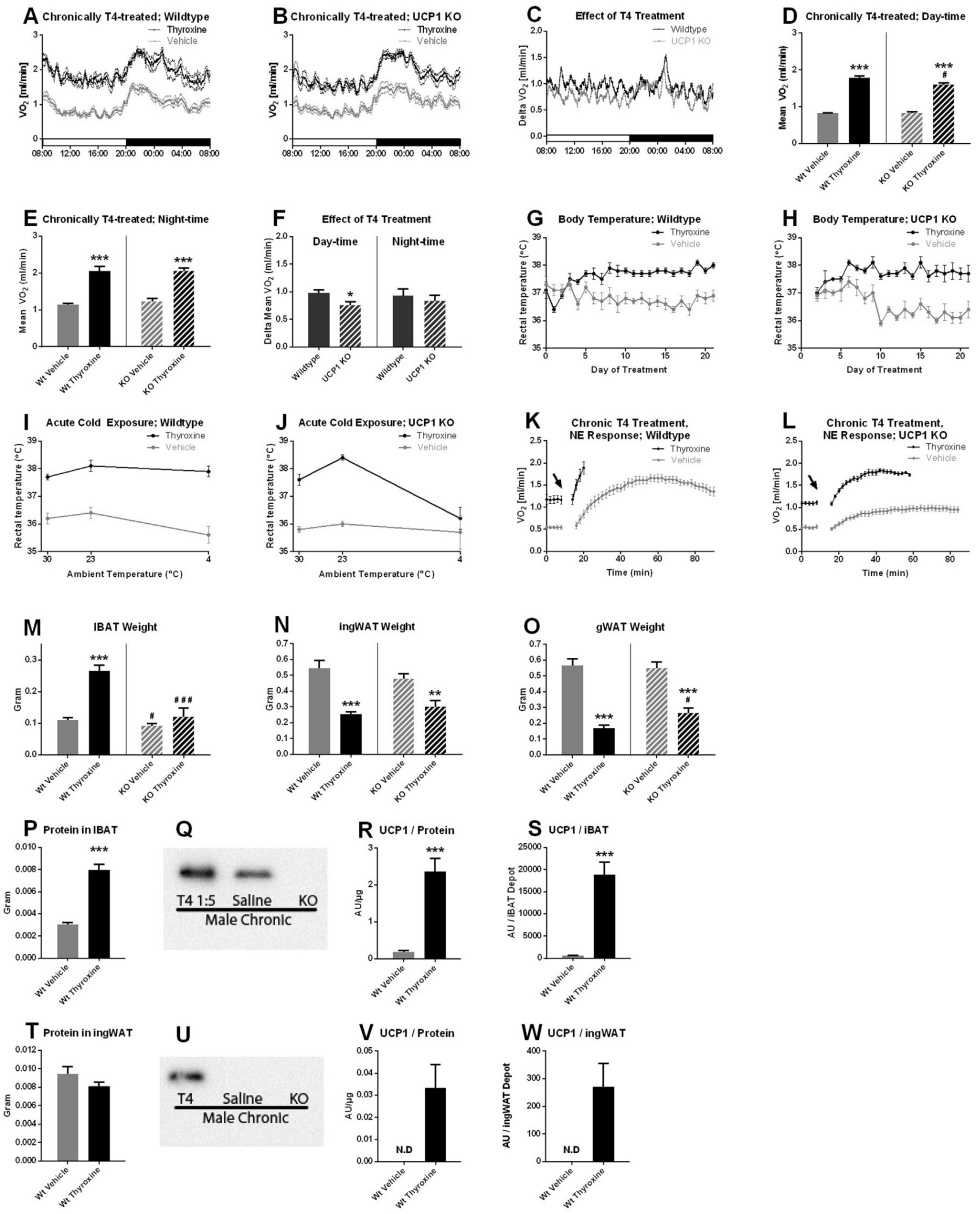
**Effects of chronic treatment of male mice with thyroxine.** Male mice were treated with thyroxine or not, similarly to the female mice described in detail above. A–F correspond to Figure 2 AF; G–H correspond to Figure 4 A–D; K and L correspond to Figure 5 F,G; M−U correspond to Figure 6 A–I and V and W are calculations for inguinal adipose tissue similar to those performed for IBAT in Figure 6F,G. N values for saline/thyroxine-treated: A: 8/5; B: 8/7; D: 8/5 8/7; E 8/5 8/7; G 8/8 (some values missing certain days); H: 4/4; I 8/8; J: 4/3; K: 5/5; L: 8/7; M−W 8/5 8/7 where relevant. All values are means ± SE. Statistics as in Figure 1.

All the above studies were performed on female mice. As it has been reported that sex-specific responses to hyperthyroidism may occur [28], a similar set of studies were performed on male mice of the same age and under otherwise similar conditions. Qualitatively very similar results were obtained in all respects (Figure 7). Particularly, the basal metabolic rate was nearly doubled (Figure 7A–F) and the body temperature was increased (Figure 7G,H). Also, in the male wildtype mice, transfer to a colder environment (23 or 4 °C) did not result in a lower body temperature (Figure 7I), supporting the notion that the increased body temperature was not a consequence of the increased metabolism, i.e. not hyperthermia due to difficulties in dissipating the heat; rather also in males it represented pyrexia: a defended, centrally regulated increase in body temperature. A quantitative difference was found in the initial levels of brown adipose tissue UCP1, with the males having levels some 3-fold higher than the females (Figure 7S versus Figure 6G), whereas the increase following chronic thyroxine treatment was very similar, being about 30-fold for both sexes. A qualitative difference was that UCP1 protein was detected in the inguinal adipose depot only of the male mice that were thyroxine-treated. The amounts, however, were minor (total amounts in the tissue were 250 AU as compared to 18000 AU in the interscapular brown adipose tissue, i.e. almost 100-fold less in the inguinal depot).

## 4 Discussion

In the present investigation, we have examined metabolic effects of thyroid hormone at thermoneutrality, thus avoiding confounding factors related to the metabolic effects of the cold stress experienced by mice exposed to standard housing conditions. We found that peripherally delivered thyroxine led to (the expected) elevations in metabolic rate, body temperature, and food intake and to a loss of body fat. Importantly, all these effects were essentially the same in UCP1-ablated mice. This demonstrates that brown adipose tissue-mediated thermogenesis is not an obligatory component of the acute thyroxine-induced increased metabolism (“thyroid thermogenesis”). Nonetheless, chronic thyroxine treatment markedly increased the levels of UCP1 protein in brown adipose tissue. Thyroxine-treated mice maintained a higher body temperature than vehicle-treated mice even at very low ambient temperatures; this demonstrates that the thyroxine-treated animals defend this elevated body temperature. Therefore, the elevated body temperature is not hyperthermia (i.e. it is not a consequence of hypermetabolism occurring in the absence of a change in the centrally regulated body temperature) but a defended elevation of the body temperature, i.e. pyrexia. The data are of general relevance for discussions of the apparent phenomenon of thyroid-hormone-induced thermogenesis.

### 4.1 Thyroid hormone effects in the euthyroid to hyperthyroid transition may be principally different from thyroid hormone effects in hypothyroidism

Analysis of the effects of thyroid hormone traditionally utilizes two different experimental platforms. One is that studied here, in which euthyroid animals are treated with thyroid hormone, with effects on metabolism. The other is when animals are made hypothyroid and the effect of replenishment of thyroid hormone is studied. In the latter case, it is clearly so that the expression of numerous genes that have been repressed due to the hypothyroidism – often due to the unligated thyroid hormone receptor functioning as a repressor [31] – are augmented, with subsequent metabolic effects. This is the case both generally in the animal [2], [3] and specifically in brown adipose tissue [32]. Although it may be argued that hyperthyroidism is merely a further augmentation of the hypothyroid to euthyroid transition, this may not be the case; repression may have been fully alleviated already in the euthyroid state. The analysis of the present data, therefore, does not refer to earlier results from studies of hypothyroid animals as an explanation for the here observed metabolic effects of thyroid hormone.

Similarly, the thyroxine doses used here exceed those normally used for re-introduction of the euthyroid state in hypothyroid animals and were chosen to ensure that the thyroid hormone effects were saturated so that maximal thyroid thermogenesis was induced.

### 4.2 Higher body temperature in thyroxine-treated mice is pyrexia and not hyperthermia

Hyperthyroidism is normally associated with an increased body temperature, as also observed here (Figure 4, Figure 7G,H). This is often referred to as hyperthermia. Although this term may be used colloquially for any state of increased body temperature, thermoregulatory analysis distinguishes between hyperthermia and pyrexia (fever) (for further discussion of terminology, see Ref. [29]). Hyperthermia is an increased body temperature caused by an inability of the organism to dissipate the heat transferred to or generated within it. Pyrexia is an increase in body temperature that the organism strives for. (We prefer the term “pyrexia” rather than “fever”, due to the common association of the word “fever” with infections).

Experimentally, hyperthermia can be relieved if the organism is given the possibility to more easily dissipate the extra heat, e.g. by being placed in colder surroundings. It was clear that the body temperature of the thyroxine-treated mice studied here was not reduced when the mice were acutely transferred to lower environmental temperatures (Figure 4, Figure 7) and this was true for both milder (23 °C) and more severe (4 °C) cold conditions for the wildtype mice.

The thyroxine-treated UCP1 KO mice only maintained their elevated body temperature at 23 °C, but it was decreased at 4 °C. However, as this occurred both in the vehicle-treated and in the thyroxine-treated UCP1 KO mice, the inability to maintain the elevated body temperature was fully due to the general inability of these mice (that were acclimated to 30 °C) to defend their body temperature in acute cold [30]. Analysis of the Scholander plots (Figure 4E,F) further supports the notion that the increased body temperature is not hyperthermia, because the slopes do not coincide at lower ambient temperatures as they would if the increased body temperature had been hyperthermia. Instead, the thyroxine-treated mice utilize more energy at any ambient temperature to maintain their elevated body temperature. The result of this experiment in mice is also in accordance with the results of earlier experiments in rats [20], [33] that can be interpreted [34] to show a similar increase of 1–2 °C in defended body temperature. Thus, the conclusion from these data is that the elevated body temperature in hyperthyroid states is not a consequence of the increased thermogenesis but is an independent outcome of the hyperthyroidism.

### 4.3 Thyroid hormone directly affects body temperature control

The observation that the effect of thyroxine on body temperature is not secondary to increased metabolism is also in agreement with the result that the increased body temperature is not dependent upon the presence of UCP1 (Figure 4A,B). Body temperature is believed to be controlled primarily by the POAH (preoptic chiasma anterior hypothalamus) [35], and this effect of thyroxine should therefore be centrally mediated. Indeed, similar to what was observed here by peripheral thyroxine treatment, we have earlier observed that direct infusion of T_3_ into the lateral ventricle in the brain resulted in an increase in body temperature [6] at least in wildtype mice (and an increase in body temperature can also be observed in thyroxine-treated UCP1 KO mice in that paper). Thus, the effect of peripheral thyroxine treatment is most probably centrally mediated. As the thyroxine-induced increase in body temperature has generally been considered to be hyperthermia, studies of the effects of thyroid hormone have not generally included studies of direct effects on the central control of body temperature. There are thus no detailed investigations as to how thyroid hormone affects the POAH and of the augmentation of this effect with time.

### 4.4 “Thyroid thermogenesis” is largely independent of brown adipose tissue thermogenic activity

A major finding in this study is that acute thyroid thermogenesis is not dependent upon brown fat-derived thermogenesis (i.e. not UCP1-dependent). In general, the thyroxine-induced thermogenesis was quantitatively similar in wildtype and UCP1 KO mice, although a minor component (<25%) of the day-time induced thermogenesis was absent when the experiment was performed in UCP1 KO mice (Figure 2F). This observation may seem at first sight to be contradictory to earlier studies indicating that thyroid hormone induces nearly all its thermogenesis through a centrally mediated process involving the ventromedial hypothalamus and an increased activation of the sympathetic nerves innervating brown adipose tissue [4], [5]; indeed, daily metabolic rate was increased by centrally infused T_3_ only in wildtype and not in UCP1 KO mice [6]. However, these results were obtained with mice that had been pre-acclimated to 18 °C and thus had acquired ample brown-fat thermogenic capacity. When similar experiments were performed with 30 °C-acclimated mice, a UCP1-dependent component was not identifiable (Alvarez-Crespo et al., unpublished obs.). Thus, if the mice possess ample brown-fat thermogenic capacity, it may be activated – but the peripherally induced thyroid thermogenesis observed here occurs even in the absence of brown fat-derived thermogenesis.

### 4.5 “Thyroid thermogenesis” is independent of brite/beige adipose tissue thermogenic activity

In addition to the possibility that thyroid thermogenesis emanates from classical brown adipose tissue, the possibility has been discussed that it may be mediated by brite/beige adipose tissue. Indeed, both centrally and peripherally applied thyroid hormone has been reported to increase UCP1 in brite/beige adipose tissue, with the implication that the activity of this UCP1 would contribute to or even explain thyroid thermogenesis [6], [36], [37]. In this study, we were to detect UCP1 protein in the brite/beige adipose tissue of thyroxine-treated female mice (Figure 6I) and we only found minuscule amounts of UCP1 protein in thyroxine-treated male mice (Figure 7U–W) as compared to the amounts of UCP1 protein found in classical brown adipose tissue. However, our data demonstrate that, even if thyroid hormone may induce small amounts of UCP1 in brite/beige tissues, this UCP1 is not the molecular background for thyroid thermogenesis.

### 4.6 How does hyperthyroidism recruit brown adipose tissue?

We found that chronic thyroxine treatment led to a recruitment of brown adipose tissue (Figure 6), principally in agreement with earlier observations [20], [23], [24]. This could occur through direct or indirect effects. There are a series of studies indicating the necessity of local thyroid hormone for brown adipose tissue recruitment (e.g. [38]). However, these studies have generally investigated the transition from a hypothyroid to a euthyroid state and are therefore probably principally different from the conditions examined here, the transition from euthyroid to hyperthyroid. Also, most earlier investigations have concluded that thyroid hormone is essential for brown adipose tissue recruitment such that without thyroid hormone, norepinephrine would be unable to recruit the tissue.

The alternative would be that thyroid hormone stimulates the tissue indirectly. The studies of Lopez et al. [4] clearly indicated that thyroid hormone stimulated areas in the ventromedial hypothalamus that in their turn led to an enhanced sympathetic drive to the tissue, and this drive would with time result in a recruitment of the tissue. In the present study, some of the thyroid hormone injected would presumably pass the blood-brain-barrier and stimulate the ventromedial hypothalamus. This would thus be an indirect recruitment by thyroid hormone, mediated by the sympathetic nervous system. Whether this is the mechanism involved could be examined by severing the nerves to the brown adipose tissue and establishing whether the recruitment was still observable, as we recently did in connection with so-called diet-induced thermogenesis and its interaction with brown adipose tissue [9]. Nevertheless, the heat produced through this would be “extra” heat, i.e. heat production not demanded by the thermoregulatory system. It would need to be dissipated by the mouse to prevent true hyperthermia.

### 4.7 An increased capacity for UCP1-dependent adrenergically stimulated thermogenesis is induced by thyroxine treatment

In the chronically thyroxine-treated mice, the response to norepinephrine injection was greater than in the controls (Figure 5, Figure 7K,L). That chronic thyroxine treatment induces a higher response to norepinephrine in the wildtype mice is fully in agreement with the recruitment of brown adipose tissue observed in these mice (Figure 6, Figure 7P–S).

### 4.8 Is an increased capacity for UCP1-independent adrenergically stimulated thermogenesis induced by thyroxine treatment?

The responses to norepinephrine injection in both short-term and chronic thyroxine-treated UCP1 KO mice were higher than in the vehicle-treated mice (Figure 5, Figure 7L). This would initially seem to imply that thyroid hormone could induce extra – UCP1-independent – mechanisms for adrenergically induced nonshivering thermogenesis. Whereas this possibility cannot be excluded, an alternative explanation would be that the increased response in these cases is secondary to the higher body temperature of these mice and the ensuing increase in reaction kinetics. Thus, the higher rates would not necessitate the introduction of extra thyroxine-induced adrenergic thermogenic mechanisms but the rates would merely reflect a change in the kinetics.

### 4.9 What is the nature of “thyroid thermogenesis”?

The conclusion that thyroid thermogenesis is UCP1-dependent only to a very minor extent evidently raises the question of the nature of the main part of the increased metabolism observed during thyroxine treatment, nearly a doubling of metabolism, occurring to a significant extent even within a few days of thyroxine treatment (Figure 1, Figure 2, Figure 7A–F). The literature suggests several mechanisms for this high metabolism. Many are related to increased expression of certain enzymes, but it is unclear how this in itself would result in higher metabolic rates. Typically, an increased expression of the Na/K-ATPase is mentioned [3], but a mere increase in the amount of this enzyme would not result in an increased metabolic rate. This would only happen if also an increase in membrane permeability were induced, and this has not been unequivocally demonstrated. Similar reasoning can be applied to the sarcoplasmic Ca^2+^ pumps. Thus, there is presently no established molecular mechanism for thyroid thermogenesis; this is in contrast to the case for brown-fat-derived thermogenesis that is understandable through the activity of UCP1.

### 4.10 The relation between body temperature and metabolism

What is firmly established in the present investigation is that thyroxine induces an increase in body temperature in the order of 1–2 °C. If an organism fully behaves as a simple chemical reaction, a two-to-three fold increase in rates for every 10 °C is expected, corresponding to a 7–12% increase in metabolic rate for each degree increase in body temperature. This could then in itself explain about a fifth of the increased metabolism.

But do mammals behave simply as a chemical reaction? This is not a question that can easily be answered experimentally, as chronic pyrexic (fever) states are not easily identified (except for the hyperthyroid pyrexia studied here). Most metabolic studies of fever states have investigated acute but transient events after injection of pyrogens of different nature and they are not relevant for the present issue. However, there is a group of animals that present with a high, stable body temperature: the passerine birds. Passerine birds have a body temperature of about 40.5 °C, i.e. about 1 °C higher than non-passerine birds and 2.5 °C higher than mammals. However, their metabolism is 70% higher than that of non-passerine birds and 100% higher than that of mammals [39]. One implication of this could be that maintaining a higher (1–2 °C) body temperature is associated with a much higher increase in metabolic rate than would be predicted simply based on reaction kinetics, i.e. a kind of metabolic acceleration corresponding to the metabolic depression seen at low body temperatures conditions (torpor and hibernation) where metabolism is decreased more than expected from the decline in body temperature and simple Q_10_ effects [40], [41]. On the other hand, leptin deficiency/restorationt is associated with a ≈1 °C change in body temperature that is not associated with altered metabolic rate [42]. Still, one possibility to be considered is that the reason that no molecular mechanism for thyroid thermogenesis has as yet been established is that thyroid thermogenesis does not exist; the increased metabolism could be a consequence (and not a cause) of the increased body temperature induced by thyroid hormone.

## References

[1] J.E. Silva, The thermogenic effect of thyroid hormone and its clinical implications, Annals of internal medicine, 139 (2003) 205-213. 12899588

[2] J.E. Silva, Thermogenic mechanisms and their hormonal regulation, Physiol Rev, 86 (2006) 435-464. 10.1152/physrev.00009.2005 16601266

[3] R. Mullur, Y.Y. Liu, G.A. Brent, Thyroid hormone regulation of metabolism, Physiol Rev, 94 (2014) 355-382. 10.1152/physrev.00030.2013 PMC4044302 24692351

[4] M. Lopez, L. Varela, M.J. Vazquez, S. Rodriguez-Cuenca, C.R. Gonzalez, V.R. Velagapudi, D.A. Morgan, E. Schoenmakers, K. Agassandian, R. Lage, P.B. Martinez de Morentin, S. Tovar, R. Nogueiras, D. Carling, C. Lelliott, R. Gallego, M. Oresic, K. Chatterjee, A.K. Saha, K. Rahmouni, C. Dieguez, A. Vidal-Puig, Hypothalamic AMPK and fatty acid metabolism mediate thyroid regulation of energy balance, Nat Med, 16 (2010) 1001-1008. 10.1038/nm.2207 PMC2935934 20802499

[5] B. Cannon, J. Nedergaard, Thyroid hormones: igniting brown fat via the brain, Nat Med, 16 (2010) 965-967. 10.1038/nm0910-965 20823876

[6] M. Alvarez-Crespo, R.I. Csikasz, N. Martinez-Sanchez, C. Dieguez, B. Cannon, J. Nedergaard, M. Lopez, Essential role of UCP1 modulating the central effects of thyroid hormones on energy balance, Mol Metab, 5 (2016) 271-282. 10.1016/j.molmet.2016.01.008 PMC4812006 27069867

[7] B. Cannon, J. Nedergaard, Brown adipose tissue: function and physiological significance, Physiol. Rev., 84 (2004) 277-359. 10.1152/physrev.00015.2003 14715917

[8] J. Nedergaard, B. Cannon, Brown adipose tissue as a heat-producing thermoeffector, Handbook of clinical neurology, 156 (2018) 137-152. 10.1016/B978-0-444-63912-7.00009-6 30454587

[9] A.W. Fischer, C. Schlein, B. Cannon, J. Heeren, J. Nedergaard, Intact innervation is essential for diet-induced recruitment of brown adipose tissue, Am J Physiol Endocrinol Metab, 316 (2019) E487-E503. 10.1152/ajpendo.00443.2018 PMC6459298 30576247

[10] J.E. Silva, Physiological importance and control of non-shivering facultative thermogenesis, Frontiers in bioscience, 3 (2011) 352-371. 10.2741/s156 21196381

[11] J.L. Leonard, S.A. Mellen, P.R. Larsen, Thyroxine 5'deiodinase activity in brown adipose tissue, Endocrinology, 112 (1983) 1153-1155. 10.1210/endo-112-3-1153 6822208

[12] R. Jones, L. Henschen, N. Mohell, J. Nedergaard, Requirement of gene transcription and protein synthesis for cold- and norepinephrine-induced stimulation of thyroxine deiodinase in rat brown adipose tissue, Biochim Biophys Acta, 889 (1986) 366-373. 10.1016/0167-4889(86)90200-4 3790581

[13] P. Seale, S. Kajimura, W. Yang, S. Chin, L.M. Rohas, M. Uldry, G. Tavernier, D. Langin, B.M. Spiegelman, Transcriptional control of brown fat determination by PRDM16, Cell Metab, 6 (2007) 38-54. 10.1016/j.cmet.2007.06.001 PMC2564846 17618855

[14] E.D.R. Arrojo, T.L. Fonseca, J.P. Werneck-de-Castro, A.C. Bianco, Role of the type 2 iodothyronine deiodinase (D2) in the control of thyroid hormone signaling, Biochim Biophys Acta, 1830 (2013) 3956-3964. 10.1016/j.bbagen.2012.08.019 PMC4979226 22967761

[15] A. Matthias, K.B. Ohlson, J.M. Fredriksson, A. Jacobsson, J. Nedergaard, B. Cannon, Thermogenic responses in brown fat cells are fully UCP1-dependent. UCP2 or UCP3 do not substitute for UCP1 in adrenergically or fatty scid-induced thermogenesis, J Biol Chem, 275 (2000) 25073-25081. 10.1074/jbc.M000547200 10825155

[16] Y. Okamatsu-Ogura, K. Fukano, A. Tsubota, A. Uozumi, A. Terao, K. Kimura, M. Saito, Thermogenic ability of uncoupling protein 1 in beige adipocytes in mice, PLoS One, 8 (2013) e84229. 10.1371/journal.pone.0084229 PMC3875535 24386355

[17] K. Ganeshan, A. Chawla, Warming the mouse to model human diseases, Nature reviews. Endocrinology, 13 (2017) 458-465. 10.1038/nrendo.2017.48 PMC5777302 28497813

[18] S. Enerback, A. Jacobsson, E.M. Simpson, C. Guerra, H. Yamashita, M.-E. Harper, L.P. Kozak, Mice lacking mitochondrial uncoupling protein are cold-sensitive but not obese, Nature, 387 (1997) 90-94. 10.1038/387090a0 9139827

[19] J. Himms-Hagen, Thyroid hormones and thermogenesis, in: L. Girardier, M.J. Stock (Eds.) Mammalian Thermogenesis, Chapman and Hall, London, 1983, pp. 141-177.

[20] J. Nedergaard, A. Dicker, B. Cannon, The interaction between thyroid and brown-fat thermogenesis. Central or peripheral effects?, Ann. N.Y. Acad. Sci., 813 (1997) 712-717. 10.1111/j.1749-6632.1997.tb51772.x 9100960

[21] J. Nedergaard, B. Cannon, The browning of white adipose tissue: some burning issues, Cell Metab, 20 (2014) 396-407. 10.1016/j.cmet.2014.07.005 25127354

[22] A.V. Kalinovich, J.M. de Jong, B. Cannon, J. Nedergaard, UCP1 in adipose tissues: two steps to full browning, Biochimie, 134 (2017) 127-137. 10.1016/j.biochi.2017.01.007 28109720

[23] U. Sundin, GDP binding to rat brown fat mitochondria: effects of thyroxine at different ambient temperatures., American journal of physiology. Cell physiology, 241 (1981) C134-C139. 10.1152/ajpcell.1981.241.3.C134 7282915

[24] U. Sundin, I. Mills, J.N. Fain, Thyroid-catecholamine interactions in isolated rat brown adipocytes, Metabolism, 33 (1984) 1028-1033. 10.1016/0026-0495(84)90232-4 6092828

[25] F.a.N. Board, Dietary reference intakes for energy, carbohydrate, fiber, fat, fatty acids, cholesterol, protein, and amino acids., Natl. Acad. Press, Washington, DC (2005) 107-264. 10.1016/s0002-8223(02)90346-9 12449285

[26] V.G. Ivlev, Eine mikromethode zur Bestimmung des Kaloriegehalts von Nahrstoffen, Biochem. Z., 275 (1934) 49-55.

[27] J.B. Weir, New methods for calculating metabolic rate with special reference to protein metabolism, J Physiol, 109 (1949) 1-9. 10.1113/jphysiol.1949.sp004363 PMC1392602 15394301

[28] H. Rakov, K. Engels, G.S. Hones, K.H. Strucksberg, L.C. Moeller, J. Kohrle, D. Zwanziger, D. Fuhrer, Sex-specific phenotypes of hyperthyroidism and hypothyroidism in mice, Biology of sex differences, 7 (2016) 36. 10.1186/s13293-016-0089-3 PMC4995626 27559466

[29] C.J. Gordon, A review of terms for regulated vs. forced, neurochemical-induced changes in body temperature, Life sciences, 32 (1983) 1285-1295. 10.1016/0024-3205(83)90802-0 6339853

[30] V. Golozoubova, E. Hohtola, A. Matthias, A. Jacobsson, B. Cannon, J. Nedergaard, Only UCP1 can mediate adaptive nonshivering thermogenesis in the cold, FASEB J., 15 (2001) 2048-2050. 10.1096/fj.00-0536fje 11511509

[31] V. Golozoubova, H. Gullberg, A. Matthias, B. Cannon, B. Vennstrom, J. Nedergaard, Depressed thermogenesis but competent brown adipose tissue recruitment in mice devoid of all hormone-binding thyroid hormone receptors, Molecular endocrinology, 18 (2004) 384-401. 10.1210/me.2003-0267 14630998

[32] M.J. Obregon, Adipose tissues and thyroid hormones, Frontiers in physiology, 5 (2014) 479. 10.3389/fphys.2014.00479 PMC4263094 25566082

[33] M. Szekely, Effects of thyroxine treatment of different duration on oxygen consumption and body temperature at different ambient temperatures in the rat, Acta Physiol. Acad. Sci. Hung., 37 (1970) 51-55. 5433559

[34] B. Cannon, J. Houstek, J. Nedergaard, Brown adipose tissue. More than an effector of thermogenesis?, Ann. N. Y. Acad. Sci., 856 (1998) 171-187. 10.1111/j.1749-6632.1998.tb08325.x 9917877

[35] S.F. Morrison, Central control of body temperature, F1000Research, 5 (2016). 10.12688/f1000research.7958.1 PMC4870994 27239289

[36] N. Martinez-Sanchez, J.M. Moreno-Navarrete, C. Contreras, E. Rial-Pensado, J. Ferno, R. Nogueiras, C. Dieguez, J.M. Fernandez-Real, M. Lopez, Thyroid hormones induce browning of white fat, J. Endocrinol. , 232 (2017) 351-362. 10.1530/JOE-16-0425 PMC5292977 27913573

[37] J. Weiner, Thyroid hormones and browning of adipose tissue, Mol. Cell. Endocrinol. , 458 (2017) 156-159. 10.1016/j.mce.2017.01.011 28089823

[38] A.C. Bianco, J.E. Silva, Intracellular conversion of thyroxine to triiodothyronine is required for the optimal thermogenic function of brown adipose tissue, J. Clin. Invest., 79 (1987) 295-300. 10.1172/JCI112798 PMC424048 3793928

[39] T.J. Dawson, A.J. Hulbert, Standard metabolism, body temperature, and surface areas of Australian marsupials, Am J Physiol, 218 (1970) 1233-1238. 10.1152/ajplegacy.1970.218.4.1233 5435424

[40] K.B. Storey, J.M. Storey, Metabolic-rate depression and biochemical adaptation in anaerobiosis, hibernation and estivation, Quart. Rev. Biol. , 65 (1990) 145-174. 10.1086/416717 2201054

[41] M. Jastroch, S. Giroud, P. Barrett, F. Geiser, G. Heldmaier, A. Herwig, Seasonal Control of Mammalian Energy Balance: Recent Advances in the Understanding of Daily Torpor and Hibernation, J. Neuroendocrinol., 28 (2016). 10.1111/jne.12437 27755687

[42] A.W. Fischer, C.S. Hoefig, G. Abreu-Vieira, J.M.A.de Jong, N. Petrovic, J. Mittag, B. Cannon and J.Nedergaard, Leptin raises defended body temperature without activating thermogenesis, Cell Reports 14, 2016, 1621-1631. 10.1016/j.celrep.2016.01.041 26876182

